# Generating Engagement on the Make Healthy Normal Campaign Facebook Page: Analysis of Facebook Analytics

**DOI:** 10.2196/11132

**Published:** 2019-01-14

**Authors:** James Kite, Anne Grunseit, Vincy Li, John Vineburg, Nathan Berton, Adrian Bauman, Becky Freeman

**Affiliations:** 1 Prevention Research Collaboration Sydney School of Public Health and Charles Perkins Centre The University of Sydney The University of Sydney Australia; 2 The Australian Prevention Partnership Centre The University of Sydney Australia; 3 New South Wales Office of Preventive Health Liverpool Australia; 4 Centre for Population Health New South Wales Ministry of Health North Sydney Australia; 5 Strategic Communications and Engagement New South Wales Ministry of Health North Sydney Australia

**Keywords:** social media, Facebook, overweight and obesity, mass media campaign, evaluation

## Abstract

**Background:**

Facebook is increasingly being used as part of mass media campaigns in public health, including the Make Healthy Normal (MHN) campaign in New South Wales, Australia. Therefore, it is important to understand what role Facebook can play in mass media campaigns and how best to use it to augment or amplify campaign effects. However, few studies have explored this.

**Objective:**

This study aimed to investigate usage of and engagement with the MHN Facebook page and to identify influential factors in driving engagement with the page.

**Methods:**

We examined both post-level and page-level analytic data from Facebook from the campaign’s launch in June 2015 to September 2017. For post-level data, we conducted a series of negative binomial regressions with four different outcome measures (likes, shares, comments, post consumers), including some characteristics of Facebook posts as predictors. We also conducted time series analyses to examine associations between page-level outcomes (new page likes or “fans” and number of engaged users) and different measures of exposure to the page (number of unique users reached and total count of impressions) and to television advertising.

**Results:**

Of the 392 posts reviewed, 20.7% (n=81) received a paid boost and 58.9% (n=231) were photo posts. We found that posts that received a paid boost reached significantly more users and subsequently received significantly more engagement than organic (unpaid) posts (*P*<.001). After adjusting for reach, we found the effect of being paid was incremental for all outcome measures for photos and links, but not videos. There were also associations between day of the week and time of post and engagement, with Mondays generally receiving less engagement and posts on a Friday and those made between 8 AM and 5 PM receiving more. At the page level, our time series analyses found that organic impressions predicted a higher number of new fans and engaged users, compared to paid impressions, especially for women. We also found no association between television advertising and engagement with the Facebook page.

**Conclusions:**

Our study shows that paying for posts is important for increasing their reach, but that page administrators should look to maximize organic reach because it is associated with significantly higher engagement. Once reach is accounted for, video posts do not benefit from being paid, unlike the other post types. This suggests that page administrators should carefully consider how they use videos as part of a Facebook campaign. Additionally, the lack of association between television advertising and engagement suggests that future campaigns consider how best to link different channels to amplify effects. These results highlight the need for ongoing evaluation of Facebook pages if administrators are to maximize engagement.

## Introduction

### Background

Facebook is the largest social media platform in the world, with more than 1.4 billion daily users on average in December 2017 [1]. In Australia, nearly two-thirds of adults have a Facebook profile, making it the most popular social media platform in the country [2]. It is also the most intensely used social media platform; around 40% of Australian Facebook users log in 20 times or more per week. Further, Facebook is one of the most commonly used social media platforms for engaging with health issues [3]. It is no surprise then that public health organizations are using Facebook to communicate their messages, either as stand-alone campaigns or as an additional channel in a broader mass media campaign [4,5]. In both cases, organizations are seeking to capitalize on the wide reach of Facebook, the ability to engage directly with their target audiences, and the potential for generating marketing directly between consumers (word-of-mouth marketing), which lends credibility to a brand and is known to be one of the most trusted forms of marketing [6-8]. Within mass media campaigns specifically, the intention is that Facebook posts will augment or amplify campaign messages and, in so doing, increase the impact of the campaign [9].

The theory behind Facebook use for public health communication places “engagement” as a critical first step in achieving change. Creating engagement, defined as users “liking,” sharing, commenting, or clicking on any content, is important for two main reasons: it demonstrates that the content is attention grabbing and it directly influences the reach of the content and of future content through the Facebook algorithm [10]. The algorithm determines the amount of exposure a post receives and to whom it is shown, although it should be noted that Facebook has revealed little on the specific parameters it uses to prioritize posts. However, what is clear is that the characteristics of the post and the engagement it receives are factors in the algorithm’s calculations [4], making it essential to investigate what drives engagement in order to maximize Facebook’s marketing potential for public health campaigns. Facebook also allows page administrators to pay to increase the reach of a post, making it important to investigate the interaction between paying for posts and other post characteristics.

Despite the potential of Facebook and other social media for public health and health communication being well recognized [11-14], there is limited evidence available to guide practice. The evidence we do have is often either descriptive or based on small-scale trials [5,15-18], with suggestive but modest evidence that social media can be effective in changing health outcomes [19,20]. How to build engagement with health content on Facebook has been recognized as one area in particular need of more evidence given the role it plays in the theory of health communication on social media [21]. Currently, there is some evidence that testimonials, positive emotional appeals, and informative posts are associated with higher engagement, whereas posts that evoke negative emotions, use conventional marketing techniques (eg, sponsorships), or are posted during or after work hours are associated with lower engagement [4,22-24]. Similarly, posts that use photos and videos appear to generate higher engagement, although this is most likely due to the Facebook algorithm preferencing such content over other post types. In addition, one study that examined 20 public health Facebook pages covering a range of health issues speculated that particular health issues may be more suitable to Facebook [4]. However, they lamented that they were unable to test this, highlighting it as an area worthy of further research.

In addition, the available evidence has limited relevance to mass-reach campaigns, creating the risk that social marketers will use Facebook without considering what strategy they should employ to best use the platform in a broader campaign [25,26]. It is therefore important to investigate associations between Facebook engagement and traditional communication channels such as television. To our knowledge, no study has examined these associations. The evaluation of the Tips From Former Smokers (Tips) antismoking campaign in the United States did provide some insights into the relationship between online and traditional television marketing for public health purposes, although how relevant this is to Facebook is uncertain. Tips showed an association between television advertising and online behaviors, including increased visits to the campaign website and other cessation-related websites and searches for cessation information [27,28]. The evaluation also found that digital video was more cost-efficient at generating awareness compared to television, although the authors note that television advertising is still important because it reaches more people [29]. Another study compared the cost-effectiveness of three media formats (television, online video, and online display advertising) for delivering an antismoking campaign [30]. This study found that online display advertising was the most cost-effective way of achieving Web page views, calls to the Quitline, online registrations for a cessation support service, and requests for the smoking cessation information pack. This was followed by a combination of online video and online display, with television alone the least cost-effective. Collectively, these studies suggest that online media present a potentially useful contribution to the reach and effectiveness of antismoking campaigns, but its role in other campaigns is yet to be explored.

To our knowledge, no population-level mass media campaign has reported specifically on their use of Facebook for public health purposes. Such information is only going to become more valuable as media consumption habits are changing rapidly [31], creating questions about the accuracy of conventional wisdom on “what works” in mass media campaigns. It will also help to understand how to optimize the use of Facebook as part of a wider mass media campaign. Here we report an evaluation of the Facebook page component of an obesity prevention lifestyle campaign, Make Healthy Normal (MHN).

### The Make Healthy Normal Campaign

The MHN campaign was launched in New South Wales (NSW), Australia, in 2015, with the aim of challenging the normalization of being unhealthy and promoting physical activity, healthy eating, and healthy weight. The campaign initially targeted all adults but focused on parents with children aged 5 to 12 years and men aged 35 to 54 years from May 2017. The bulk of the advertising expenditure was directed toward television, but the campaign also made use of other channels, including Facebook. More details on the campaign are available elsewhere [32]. Briefly, the campaign was centered on two television commercials that juxtaposed unhealthy and healthy choices relating to nutrition and physical activity, while also making use of a number of other support channels, of which Facebook was one. The television commercials and most other campaign materials included the MHN website address but did not mention the Facebook page.

The MHN Facebook page had, at the time of writing, posted more than 400 times, generating over 100,000 likes, comments, and shares, and had over 32,000 page “likes” (hereafter “fans”). The page style is intended to be conversational and supportive, highlighting easy ways to eat healthier and increase physical activity, and promoting relevant NSW Government programs. The page uses both paid and organic posts (ie, content that is and is not paid advertising). The Ministry employed a strategy of paying for boosts on all posts during a specific period, as opposed to selectively boosting some posts and not others. This decision was based largely on practical considerations, especially the availability of funding.

This study aimed to investigate usage of and engagement with the MHN Facebook page as part of a broader multichannel campaign since its inception in 2015. Our research questions were: (1) What post characteristics influence the level of engagement a post receives and to what extent? (2) What page-level factors influence the number of fans, the characteristics of fans, and the engagement of fans with the MHN page over time? and (3) Is there a relationship between television advertising for the broader campaign and page-level engagement?

## Methods

### Study Overview

Facebook provides analytics (called “Insights”) to page administrators to help them monitor and understand usage of their page. In this study, we analyzed the Insights data for the MHN page since June 2015 (when the campaign launched) through to September 2017. This study was approved by the University of Sydney’s Human Research Ethics Committee (protocol number: 2017/145).

### Measures

#### Post-Level Data

We explored the characteristics of posts and their associations with engagement metrics (Table 1). Characteristics of posts included the post type, the date and time of the post, whether the post included a paid boost (paid posts tend to have a much greater increase in their reach), and the targeted behaviors. We also coded the content of the post using a modified version of the communication technique code frame developed in an earlier study [4]. The code frame was modified by collapsing some categories due to the relatively small number of posts compared to the original study. Engagement metrics were operationalized through the number of likes, shares, comments, and post consumers. Although likes technically include other Facebook “reactions” (eg, “love” and “haha”), we refer to this metric as “likes” because reactions were only introduced by Facebook a year into the campaign and the number of other reactions per post after that time was very low, typically zero.

Communication technique and target behavior were coded manually. Two coders independently coded each post, with interrater agreement for the communication techniques and target behaviors of 70% and 91%, respectively. Differences were resolved by discussion or referral to a third coder.

#### Page-Level Data

We used page-level data to examine the associations between the number of fans, the characteristics of fans, and the engagement of fans with campaign activity using the measures described in Table 2. Campaign activity was operationalized through weekly page impressions, separated by whether they were paid or organic, and weekly Target Audience Rating Points (TARPs). TARPs are an estimate of reach and frequency of exposure to television advertising, which is calculated by an external television ratings agency.

**Table 1:** Post-level measures and descriptions.

Variable		Description
Day of post		Day of the week the post first appeared
Time of post		Time post first appeared
Type		Whether the post is a photo, video, link, or text only
Paid/organic		Whether the post received a paid boost to its reach (“paid”) or not (“organic”)
Reach		The total number of unique users to whom the post was shown. Available in aggregate, as well as broken down by paid and organic reach
Consumers		The total number of unique users who clicked anywhere on the post
Likes		The number of “likes” and other “reactions” on a post. These are simple methods for users to indicate their response to a post, including to “like” the post, as well as other emotional reactions, including “love,” “haha,” “wow,” “sad,” and “angry”
Comment		The number of user comments (excluding replies) on the post
Share		The number of shares a post receives. The “share” button allows users to share the content with their Facebook friends
**Communication technique**		
	Informative	Provides information on a health issue, its associated behaviors, and/or associated consequences or benefits
	Call-to-action/instructive	Either provides instruction on how to do a behavior or encourages users to undertake a specific action (eg, call a helpline, make an appointment, register for a program or event). These were given coding precedence over informative messages
	Emotional	Aims to elicit positive (eg, hope, excitement) or negative (eg, fear) emotions in users. Also includes posts that aim to generate a positive feeling about the brand. Emotional appeals took coding precedence over informative and call-to-action/instructive, reflecting evidence that emotive content is more powerful than nonemotive content [33]
**Target behavior**		
	Eat	Information and encouragement to eat healthy food portions
	Drink	Information and encouragement to make water the drink of choice and decrease sugar-sweetened beverage consumption
	Act	Information and encouragement to be active daily and increase movement
	Other	Posts that did not relate explicitly to one of the above categories, including changes to the profile picture and page banner image and posts that shared stories about fans and stakeholders

**Table 2:** Page-level measures and descriptions.

Variable	Description
Weekly new fans	The number of new page likes per week, overall and by gender
Weekly engaged users	The number of unique users who have engaged with the page per week, overall and by gender. This includes any click on the page or one of its post or any story^a^ created by users
Weekly viral reach	The number of unique users who saw MHN or one of its posts from a story shared by a Facebook friend
Weekly paid impressions	Number of times a sponsored story or ad pointing to the page appeared in users’ News Feeds^b^. These impressions can be for fans and nonfans
Weekly organic impressions	Number of times MHN posts were displayed in News Feeds or on visits to the page. These impressions can be for fans and nonfans
Target Audience Rating Points (TARPs)	An estimate of the reach (how many people were exposed) and frequency (how often they were exposed) of the MHN television commercials per week, provided by an external ratings agency. This was used as an indicator of campaign advertising outside of Facebook

^a^A user creates a “story” by liking the page, posting to the page’s timeline, liking, commenting on, or sharing one of the page’s posts, answering a question posted by the page, responding to an event, mentioning the page, or tagging the page in a photo.

^a^News Feed refers to the constantly updating list of stories in the middle of a user’s home page, including status updates, photos, videos, links, app activity, and likes from friends, pages, and groups that they follow.

### Statistical Analysis

#### Post-Level Data

We conducted independent samples *t* tests to compare the means of engagement metrics (reach, likes, shares, comments, and post consumers) between paid and organic posts. In addition, we conducted a series of (separate) negative binomial regressions (the data were overdispersed), generating incidence rate ratios (IRRs) with the count of likes, comments, shares, and post consumers as the outcome variables, and post type, communication technique, and target behavior as categorical independent variables. The reference category for post type was photos (as this was the most populous category) and for communication technique was call-to-action/instructive because it represented a concrete action for users to take, as opposed to the other categories, which aimed to either inform or evoke emotion. For post day, each day was compared to the grand mean of all days, and for time of post during the day (8 am-5 pm; the most populous category) was used as the reference category. “Other” was used as the reference category for target behavior because these posts did not relate to specific behaviors. To examine whether the post being organic or paid interacted with other characteristics of the post, we entered two-way interaction terms for all covariates with paid/organic. Only significant two-way interactions were retained to generate the most parsimonious model. All models controlled for users’ exposure to the post by including an exposure or “offset” variable to estimate engagement with a post (ie, likes, comments) while accounting for the number of people each post was delivered to [34]. The relationship between post characteristics and engagement therefore becomes a rate per person reached.

#### Page-Level Data

To examine engagement with the MHN page over time as opposed to individual posts, we conducted time series analyses with page analytics. Time series analysis was used to account for the likely autocorrelation between observations (weekly counts) as Facebook users can view and react to content over an extended time. Further, prior engagement with content is a factor in the Facebook algorithm. Separate models were conducted for (1) new likes of the MHN page and (2) the number of unique users who “engaged” with the page for all users and for female and male users separately. In this context, “engagement” included any click on the MHN page or one of its posts or any “story” created, which would include actions such as liking the page; posting to the page’s timeline; liking, commenting on, or sharing a post; mentioning the page in one of their own posts; or tagging the page in a photo.

In addition to lag terms, each model initially included paid impressions, organic impressions, viral reach, a term for trend, and the number of TARPs as predictors. Paid impressions, organic impressions, and viral reach were rescaled to the change in the outcome variable per 10,000 because the mean weekly counts were 167,857, 29,750, and 16,781, respectively. We used backward elimination (threshold of variable retention of *P*=.10). Modeling was preceded by tests for stationarity (Dickey-Fuller and Phillips-Perron) to ensure time series modeling was appropriate [35]. We examined autocorrelation with q tests and correlograms for each model [36].

To capture the impact of changing the post content in May 2017 to target men aged 35 to 54 years and families with children aged 5 to 12 years (operationalized as women aged 25-54 years), we conducted two interrupted time series (ITS) analyses with these subpopulations only, with weekly engaged users as the outcome. The same procedure as previously described was followed for the ITS analyses, only two terms were added to the models; namely, level change and change in trend [37]. These terms and the overall trend term were retained in the final models to examine whether there were significant effects of the change in campaign approach adjusted for other significant covariates.

Post- and page-level analyses were conducted using SPSS version 22.0 (*t* tests) and Stata version 15.0 (negative binomial regression, time series, and ITS analyses).

## Results

### Post-Level Data

In total, MHN posted 392 times during our analysis period, with 20.7% (n=81) of those posts receiving a paid boost (Table 3). The majority of posts (58.9%, n=231) were photos, whereas none were text only.

Posts that received a paid boost reached significantly more users and received significantly more likes, shares, comments, and post consumers than organic posts (Table 4). Across all measures, paid posts received at least 18 times the engagement compared to organic posts.

**Figure 1:**
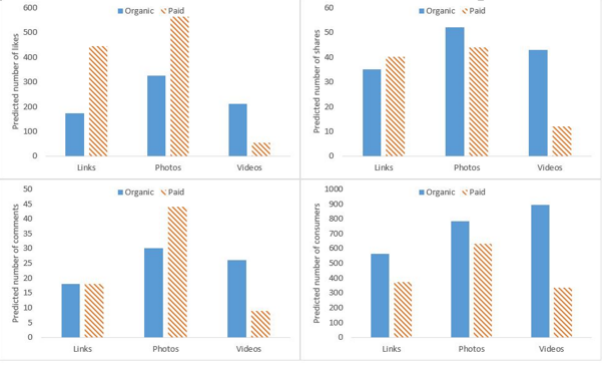
Predicted number of likes, shares, comments, and consumers by paid/organic status and post type, adjusting for reach. Note: marginal means calculated for post type by paid/organic (mean values for other covariates) based on negative binomial regressions presented in Table 5.

The significant interaction (*P*<.001) between organic/paid and post type indicated that the effect of paying was not the same across the three different types of posts (Table 5). Specifically, there was an incremental effect on likes, shares, comments, and post consumers for photos and links, but not for videos once adjusted for reach. For example, after adjusting for reach, both photo and link posts were predicted to receive more likes when paid (563 compared to 325 and 445 compared to 172, respectively), whereas paid video posts were predicted to receive only 53 likes compared to 211 for organic videos (Figure 1). A similar pattern was evident for all other engagement outcomes.

**Table 3:** Frequencies of post characteristics (N=392).

Post characteristic		Frequency, n (%)
**Paid or organic**		
	Paid	81 (20.7)
	Organic	311 (79.3)
**Communication technique**		
	Instructive/call-to-action	204 (52.0)
	Emotional	133 (33.9)
	Informative	55 (14.0)
**Post day**		
	Sunday	36 (9.2)
	Monday	51 (13.0)
	Tuesday	69 (17.6)
	Wednesday	56 (14.3)
	Thursday	69 (17.6)
	Friday	60 (15.3)
	Saturday	51 (13.0)
**Post type**		
	Photo	231 (58.9)
	Link	69 (17.6)
	Video	92 (23.5)
**Target behavior**		
	Act	118 (30.1)
	Drink	67(17.1)
	Eat	139 (35.5)
	Other	68 (17.3)
**Post time**		
	6 am to 8 am	111 (28.3)
	8 am to 5 pm	202 (51.5)
	After 5 pm	79 (20.2)

**Table 4:** Comparison of mean engagement for paid and organic posts using independent sample *t* tests.

Engagement metric	Paid mean (SD)	Organic mean (SD)	Mean difference (95% CI)	*P* value
Reach	107,764 (176,267)	3115 (2448)	104,649 (85,062-124,235)	<.001
Likes	886 (1175)	32 (33)	854 (723-985)	<.001
Shares	109 (205)	6 (8)	103 (80-126)	<.001
Comments	88 (137)	4 (6)	84 (68-99)	<.001
Consumers	1891 (3257)	86 (104)	1805 (1442-2167)	<.001

**Table 5:** Associations between post characteristics and engagement metrics per person reached calculated using negative binomial regressions adjusted for post reach.

Post characteristic			Likes, IRR^a^ (95% CI)	Shares, IRR (95% CI)	Comments, IRR (95% CI)	Post consumers, IRR (95% CI)
**Paid or organic**						
	Organic		Ref^b^	Ref	Ref	Ref
	Paid		1.51 (1.17, 1.97)	0.84 (0.64, 1.09)	1.46 (1.05, 2.03)	1.02 (0.74, 1.39)
**Post type**						
	Photo		Ref	Ref	Ref	Ref
	Link		0.53 (0.44, 0.64)	0.67 (0.52, 0.86)	0.61 (0.44, 0.84)	0.72 (0.59, 0.88)
	Video		0.65 (0.52, 0.81)	0.84 (0.63, 1.11)	0.85 (0.60, 1.21)	1.14 (0.91, 1.43)
**Post day^c^**						
	Sunday		0.93 (0.75, 1.15)	0.90 (0.69, 1.18)	0.92 (0.65, 1.29)	0.93 (0.74, 1.16)
	Monday		0.73 (0.61, 0.88)	0.64 (0.51, 0.81)	0.81 (0.61, 1.09)	0.72 (0.60, 0.87)
	Tuesday		1.06 (0.91, 1.23)	1.18 (0.98, 1.42)	0.90 (0.70, 1.14)	0.83 (0.71, 0.98)
	Wednesday		1.00 (0.84, 1.18)	0.90 (0.73, 1.11)	1.01 (0.77, 1.33)	2.01 (1.67, 2.43)
	Thursday		1.01 (0.87, 1.18)	1.15 (0.95, 1.39)	0.96 (0.75, 1.22)	0.88 (0.75, 1.03)
	Friday		1.21 (1.02, 1.43)	1.08 (0.87, 1.35)	1.33 (1.01, 1.75)	1.08 (0.91, 1.29)
	Saturday		1.05 (0.88, 1.25)	1.14 (0.91, 1.41)	1.14 (0.87, 1.49)	0.94 (0.78, 1.13)
**Time of post**						
	8 am to 5 pm		Ref	Ref	Ref	Ref
	6 am to 8 am		0.68 (0.57, 0.81)	0.91 (0.74, 1.12)	0.69 (0.53, 0.90)	0.62 (0.52, 0.74)
	After 5 pm		0.72 (0.58, 0.89)	0.91 (0.72, 1.14)	0.85 (0.64, 1.13)	0.61 (0.50, 0.73)
**Communication technique**						
	Instructive/call-to-action		Ref	Ref	Ref	Ref
	Emotional		1.18 (1.00, 1.39)	1.00 (0.81, 1.24)	0.58 (0.45, 0.75)	1.03 (0.84, 1.27)
	Informative		1.05 (0.85, 1.30)	0.90 (0.69, 1.17)	1.00 (0.72, 1.41)	0.98 (0.77, 1.24)
**Target behavior**						
	Other		Ref	Ref	Ref	Ref
	Act		0.87 (0.69, 1.08)	1.23 (0.92, 1.64)	1.11 (0.77, 1.58)	0.36 (0.28, 0.45)
	Drink		0.96 (0.73, 1.25)	1.53 (1.09, 2.15)	0.84 (0.56, 1.27)	0.32 (0.24, 0.42)
	Eat		0.81 (0.64, 1.01)	1.14 (0.85, 1.53)	0.92 (0.65, 1.31)	0.47 (0.36, 0.60)
**Interactions with paid or organic^d^**						
	**Post type**					
		Paid link	1.49 (0.98, 2.26)	1.37 (0.83, 2.25)	0.69 (0.36, 1.29)	0.83 (0.53, 1.28)
		Paid video	0.15 (0.09, 0.23)	0.32 (0.19, 0.53)	0.25 (0.13, 0.48)	0.46 (0.29, 0.74)
	**Time of post**					
		Paid 6 am to 8 am	1.45 (0.94, 2.24)	NS^e^	NS	NS
		Paid after 5 pm	1.62 (1.06, 2.48)	NS	NS	NS
	**Paid or organic/communication technique interaction**					
		Paid emotional	NS	NS	NS	0.64 (0.43, 0.96)
		Paid informative	NS	NS	NS	0.62 (0.34, 1.10)

^a^IRR: incident rate ratio.

^b^Ref: reference category.

^c^Post day is in comparison to the mean of all days.

^d^Only two-way interactions that were significant for at least one outcome are shown. Where the overall test of the interaction was nonsignificant, it was dropped from the final model.

^e^NS: nonsignificant.

Posts made on Monday received 27% fewer likes, 36% fewer shares, and 28% fewer post consumers compared to the mean, whereas posts on a Tuesday received 17% fewer post consumers. On the other hand, posts on a Wednesday received more post consumers and posts on a Friday received more likes and shares. A significant interaction (*P*=.045) between organic/paid and time of post indicated that paying for posts before 8 am and after 5 pm had a greater incremental effect on likes than paying for posts between those hours. Posts made before 8 am received fewer comments and post consumers compared to posts made between 8 am and 5 pm irrespective of whether the post was paid or organic (ie, the interaction was nonsignificant). Similarly, posts made after 5 pm received fewer post consumers. The communication technique did not influence likes, shares, and comments, with the exception of emotional posts receiving fewer comments than instructive/call-to-action posts. However, the effect of paying for a post on post consumers differed across the three different types of communication techniques (*P*=.049), such that the effect was decremental on emotive posts but not for information posts relative to instructive/call-to-action posts. Finally, drink posts received significantly more shares compared to other posts (by 53%), but act, drink, and eat posts all received between 53% and 68% fewer post consumers compared to other posts.

### Page-Level Data

Final time series models for all outcomes included only paid impressions, organic impressions, and viral reach, with all other initially included variables nonsignificant. There were three exceptions to this: organic impressions were nonsignificant in the model predicting weekly engaged male users, viral reach was nonsignificant in the model predicting weekly engaged female users, and TARPs was marginal (*P*=.07) in the model for engaged female users (Table 6).

In all models except weekly engaged males, organic impressions predicted a higher number of new fans and engaged users, compared to paid impressions. Viral reach similarly predicted a higher number of new fans and engaged users compared to paid impressions, but usually not as high as organic impressions. Organic impressions, compared to paid impressions, were considerably more influential for female users than for male users.

For the ITS analyses, none of the trend variables were significant in any of the models (Table 7). As may be expected given that the change in campaign strategy did not seem to change the trend in engagement either acutely or over time, the effect of paid and organic impressions and viral reach were similar in these subgroups to that seen in the models with the full sample and not including these trend terms.

**Table 6:** Time series results (beta coefficients with 95% CI) showing significant factors in the number of new weekly fans and engaged users (overall and by gender).

Per 10,000...	Weekly new fans, β (95% CI)			Weekly engaged users, β (95% CI)		
	Overall	Male	Female	Overall	Male	Female
Paid impressions	8.81 (7.55, 10.09)	1.97 (1.72, 2.21)	6.40 (5.38, 7.42)	68.53 (54.94, 82.12)	7.51 (6.67, 8.35)	12.67 (7.68, 17.67)
Organic impressions	58.02 (45.30, 70.74)	5.72 (2.70, 8.75)	58.82 (48.74, 68.90)	337.06 (212.22, 461.90)	NS	225.86 (160.47, 291.25)
Viral reach	22.05 (15.42, 28.67)	4.75 (3.19, 6.31)	11.02 (4.47, 17.56)	394.27 (241.86, 546.68)	28.69 (22.83, 34.57)	NS
TARPs^a^	NS^b^	NS	NS	NS	NS	–1.97 (–4.06, 0.12)

^a^TARPs: Target Audience Rating Points.

^b^NS: nonsignificant.

**Table 7:** Interrupted time series results showing significant factors in the number of new weekly fans and engaged users (by gender).

Predictors	Weekly new fans, β (95% CI)		Weekly engaged users, β (95% CI)	
	Male aged 35-54	Female aged 25-54	Male aged 35-54	Female aged 25-54
Per 10,000 paid impressions	0.96 (0.49, 1.43)	4.75 (4.05, 5.46)	3.54 (1.80, 5.28)	7.93 (5.41, 10.44)
Per 10,000 organic impressions	NS^a^	21.05 (13.20, 28.91)	NS	99.91 (69.04, 130.78)
Per 10,000 viral reach	4.84 (2.60, 7.08)	12.33 (8.12, 16.55)	25.33 (16.79, 33.87)	31.13 (8.03, 54.23)
Overall trend	–0.01 (–1.32, 1.31)	–0.08 (–8.13, 9.38)	0.19 (–5.55, 5.94)	1.30 (–3.56, 6.16)
Trend change	–0.69 (–5.81, 4.43)	0.63 (–8.13, 9.38)	–11.32 (–33.95, 11.30)	12.12 (–55.73, 79.98)
Level change	20.03 (–76.55, 116.63)	–67.91 (–150.34, 14.52)	99.08 (–297.01, 495.17)	–423.72 (–978.75, 131.31)

^a^NS: nonsignificant.

## Discussion

### Principal Findings

This study examined usage of and engagement with the MHN Facebook page, identifying influential factors at both the post and page level. We found that paying for posts significantly increases reach of posts, but that the effect was not the same across post characteristics, most notably post type. At the same time, we found that organic impressions predicted higher engagement with the MHN page compared to paid impressions, particularly for female users. Together, these findings provide an important insight into the relative value of paid and organic posts: paying for posts is useful in increasing the reach of a page but the content itself must be engaging to capitalize on word-of-mouth marketing through organic reach. In addition, we found no association between television advertising and engagement with the page, suggesting that future campaigns should consider the role of Facebook within broader mass media campaigns and how different channels can complement one another to amplify campaign effects.

Our post-level results showed that paying for posts dramatically increased their reach. This is important because, as the hierarchy of effects predicts, exposure to a message is the first step in bringing about the desired change in behavior [38]. However, the time series analyses clearly showed that organic impressions and viral reach were of critical importance in driving engagement, especially among women. This is likely due to the very high-level of trust placed in peer-to-peer communication [7] and that women are more likely to engage with health on social networking sites such as Facebook [39]. Collectively, our findings suggest that effective engagement through Facebook requires both maximizing the reach of posts through paid boosts and delivering content that users want to engage with and share in order to capitalize on word-of-mouth marketing [8]. However, how to strike a balance between the two is as yet unclear [40]. Current evidence shows that users will share content when they perceive it will be of benefit to their social network and where the risk of reputational damage is low [41], but what makes public health content “sharable” needs further investigation. This includes understanding why these results are strongest in women.

We also found that the effect of paying for posts on engagement was not the same across the different post types. Specifically, the effect of being paid on video posts appeared to be detrimental once reach was adjusted for, unlike photo and link posts. This may be due to videos requiring more effort on behalf of the user in that they need to watch and, usually, listen for an extended period. The increased effort may then mean that users will more readily scroll past a video if it does not immediately grab their attention, especially considering they will generally react negatively to obvious advertising [42]. When coupled with the fact that Facebook seems to give preferential treatment to videos in its algorithm compared to other post types [4], this finding highlights the need to weigh this preferential treatment against potential audience resistance. Public health agencies must therefore give careful consideration of how best to use videos within their campaigns on Facebook. This is particularly important given recent changes to the Facebook algorithm, particularly a promise to prioritize content generated by friends and family (ie, organic content) [43].

Day and time of post appear to have had some influence on engagement, with posts made on Mondays generally leading to lower engagement, whereas Fridays led to higher engagement. This finding might reflect users readying themselves for the working week and for the weekend, respectively. That is, on Monday users are focusing on the “serious” tasks of work, subsequently spending less time on Facebook, whereas on Friday they are preparing for more social events and activities of the weekend, reflecting a key motivation for using social media [44]. In addition, posts made outside of working hours generally led to lower levels of engagement, which was unexpected given usage patterns show the most popular times to look at social media are first thing in the morning and in the evening [2]. It is also partly in conflict with a Canadian study that found a negative association between posts made during working hours and engagement, although that study also found a negative association between engagement and posts made after work [23]. Our finding might reflect the fact that more content from larger international markets (eg, the United States and Europe) would be posted at these times, meaning the MHN content would face more competition for users’ attention, but this would not explain the Canadian finding. Alternatively, these seemingly contradictory findings suggest that the more effective time of post might vary depending on the topic of the post.

Other post characteristics, however, appeared to be less influential. That emotional posts did not generate higher levels of engagement is of particular note and largely in line with a previous study [4]. This is surprising given that these types of messages have been shown to be more effective on other media channels [45] and are often presented as being more engaging on social media [46]. The question then is whether emotional appeals are simply not what users want when engaging with health on Facebook, page administrators are not delivering content of sufficient quality, or content is not appealing to the “right” emotions. It was also noteworthy that specific behaviors generally did not generate more (or less) engagement. The exception to this was drink posts receiving more shares, suggesting that users find this content to be more novel, relevant, and interesting [47]. Further research is needed to explore these characteristics in more detail, underscoring the importance of evaluating Facebook campaigns and disseminating the results.

With regards to the page-level analyses, we found that there was no link between Facebook engagement and television advertising, in contrast to the Tips evaluation [27,28]. This is likely because the MHN television advertisements do not specifically mention a Facebook page, but rather direct people to the MHN website that also does not invite visitors to follow the campaign on Facebook. That means that the Facebook page essentially operates independently from the other campaign elements because the only way users can find the page is by searching for it within Facebook or through incidental exposure to MHN content on Facebook. It is likely that stronger linkages between the campaign components would lead to greater engagement with the Facebook page. However, it is unclear how best to synergize the campaign components, highlighting the need for robust evaluations of all components of mass media campaigns within public health. In addition, we found no evidence that the campaign narrowing its target audience led to any changes in the demographic profile of users who engaged with the Facebook page. This might be because the change in target audience occurred late in our analysis period and more time is needed to see an effect. Alternatively, it may have been because the content did not change appreciably or did not change in the right way to appeal to the new target audience. Campaign managers must therefore consider the role of each channel within a mass media campaign so that they complement one another. Some corporate brands, for instance, use Facebook as a way to associate particular events and values with their brand, as opposed to using it simply as another channel to sell their product [48]. Comprehensive formative and process evaluation would help to address these issues and help to bring about stronger linkages between the different campaign elements. However, formative and process evaluation are frequently overlooked and underreported in campaign evaluations [49].

A major limitation of our study is that we were limited to one campaign Facebook page covering just one health issue (overweight and obesity); tests with more pages that address different health issues are needed to strengthen our findings and increase their generalizability. In addition, our results should only be considered in relation to Facebook, rather than as relevant to other social media platforms given the reasons for using different platforms varies [47,50]. Our post-level analysis was also limited by a relatively small sample size of only 392 posts; more posts would have given us greater power to detect differences between the post characteristics. Finally, our interpretation of the results is based on the assumption that generating engagement is a necessary precursor to population-level impacts but, as yet, there is little evidence available to support this assumption within public health [51]. Outside of Facebook, there is suggestive evidence that skin cancer prevention messages disseminated on Twitter increased knowledge and reduced preference for a tan [52], but the impact of social media-disseminated messaging on health otherwise remains unknown. Investigating this link should be a priority for research, especially as recent changes in media consumption habits have necessitated a rethink in the relative value of different communication channels within mass media campaigns [53].

### Conclusion

Our study shows the importance of paying to boost the reach of posts on Facebook while also demonstrating the value of maximizing organic reach, particularly in relation to videos. Therefore, page administrators should give careful consideration to their marketing strategy on Facebook as sole reliance on paid or organic posts could undercut the ability of a page to generate engagement and potentially influence health at a population level. Further, our results highlight the need for campaign managers to think strategically about the role of different campaign channels and how they can amplify and complement one another. These results also underscore the importance of ongoing evaluation of campaigns on social media, especially on Facebook where the algorithm determining who sees what, when, and how often is adjusted regularly.

## Abbreviations

IRR: incident rate ratio
ITS: interrupted time series analysis
MHN: Make Healthy Normal
NSW: New South Wales
TARPs: Target Audience Rating Points
